# Eliciting neutralizing antibodies with gp120 outer domain

**DOI:** 10.1186/1742-4690-9-S2-O11

**Published:** 2012-09-13

**Authors:** Y Qin, DP Han, K Takamoto, MW Cho

**Affiliations:** 1 Iowa State University, Ames, IA, USA; 2 Albert Einstein College of Medicine, New York, NY, USA

## Background

Although gp120 elicits strong antibody responses, it fails to induce broadly neutralizing antibodies (bnAbs). One strategy being evaluated is using immunogens based on gp120 outer domain (gp120-OD). A number of gp120-OD constructs have been reported. However, none of them have been shown to induce potent nAbs. Here, we describe gp120-OD-based immunogens that can induce potent nAbs.

## Methods

We constructed gp120, gp120-OD, and a trimeric form of gp120-OD (ODx3) based on an M group consensus sequence. Proteins were expressed in 293 cells, and their antigenic properties were evaluated by immunoprecipitation using gp120 bnAbs (b12, 2G12 and 447-52D) and by surface plasmon resonance (SPR). Rabbits were immunized and antibody responses were characterized by ELISA and neutralization assays.

## Results

All three proteins were recognized by bnAbs b12, 2G12 and 447-52D. SPR analyses indicated that b12 has lower affinity to gp120-OD compared to gp120 or ODx3, largely due to a faster dissociation rate. All immunogens induced potent nAbs against Tier 1 viruses from clades B, C and AE. Neutralizing activity against Tier 2 viruses was weaker and sporadic. The induction kinetic of nAbs by gp120-OD was slower than that for gp120 and ODx3. Although the V3 loop was a major target of nAbs, results suggested other epitopes are also targeted. A panel of about 100 rabbit mAbs was generated, two of which exhibited neutralizing activity. One of them was molecularly cloned and sequenced. It exhibited a similar neutralization profile as the immune serum. Work is in progress to identify its epitope.

## Conclusion

We have successfully generated OD-based immunogens that can induce nAbs. Although they were effective primarily against Tier 1 viruses, the breadth of neutralizing activity achieved is highly significant. Our trimeric ODx3 construct is novel and is a highly promising immunogen for further development of OD-based immunogen.

